# Phytochemical and biological studies of *Panicum antidotale* aerial parts ethanol extract supported by molecular docking study

**DOI:** 10.3389/fphar.2023.1243742

**Published:** 2024-01-04

**Authors:** Imtisal Sarwar, Muhammad Asif, Talha Jamshaid, Malik Saadullah, Hafiz Muhammad Zubair, Mohammad Saleem, Usama Jamshaid, Fadia S. Youssef, Mohamed L. Ashour, Sameh S. Elhady

**Affiliations:** ^1^ Department of Pharmacology, Faculty of Pharmacy, The Islamia University of Bahawalpur, Bahawalpur, Punjab, Pakistan; ^2^ Department of Pharmaceutics, Faculty of Pharmacy, The Islamia University of Bahawalpur, Bahawalpur, Punjab, Pakistan; ^3^ Faculty of Pharmaceutical Sciences, Government College University Faisalabad, Faisalabad, Pakistan; ^4^ Punjab University College of Pharmacy, University of the Punjab, Lahore, Pakistan; ^5^ Department of Pharmaceutics, Faculty of Pharmacy, University of Strasbourg, Strasbourg, France; ^6^ Department of Pharmacognosy, Faculty of Pharmacy, Ain-Shams University, Cairo, Egypt; ^7^ Department of Natural Products, Faculty of Pharmacy, King Abdulaziz University, Jeddah, Saudi Arabia

**Keywords:** antioxidant, drug discovery, HPLC, inflammation, molecular docking, panicum antidotale, poaceae, public health

## Abstract

*Panicum antidotale* has traditionally been used as a poultice to alleviate local inflammation and painful diseases. This study aimed to evaluate the anti-inflammatory, wound-healing, analgesic, and antipyretic potential of its ethanol extract (PAAPEE) *in vivo* for the first time. *In vitro* antioxidant assays of *Panicum antidotale* using a 2,2-diphenyl-1-picrylhydrazyl assay revealed that it showed IC_50_ of 62.50 ± 6.85 μg/mL in contrast to standard, ascorbic acid, that showed IC_50_ of 85.51 ± 0.38 μg/mL. Administration of PAAPEE at a dose of 500 mg/kg (PAAPEE-500) displayed 78.44% and 75.13% inhibition of paw edema in carrageenen and histamine-induced edema models. respectively, 6 h post-treatment compared to that of the untreated group. Furthermore, it showed 68.78% inhibition of Freund’s complete adjuvant-induced edema 21 days after treatment. It reduced the animal’s rectal temperature in the yeast-induced fever model to 99.45 during the fourth h post-treatment. It significantly inhibited abnormal writhing by 44% in the acetic acid-induced pain model. PAE-500 also showed enhancement in wound closure by 72.52% with respect to that of the untreated group on the 10th day post-treatment using the excision healing of wound model. Histopathological examination of skin samples confirmed this improvement, showing enhanced tissue architecture with minimal infiltration of inflammatory cells. High-performance liquid chromatography (HPLC) of PAAPEE revealed the presence of quercetin, gallic, *p*-coumaric, benzoic, chlorogenic, syringic, ferulic, cinnamic, and sinapic acids. Molecular docking of 5-lipoxygenase and glycogen synthase kinase-3 *β* protein indicated their potential interaction within the active sites of both enzymes. Thus, *P. antidotale* serves as an effective natural wound-healing, anti-inflammatory, analgesic, and antipyretic agent.

## 1 Introduction

Inflammation is a crucial response that helps launch specific defense actions against impending inflammation and builds natural immune responses to foreign stimuli (Davies et al., 2017; Thabet et al., 2018b). Inflammation, characterized by tissue swelling, redness, and discomfort, can be triggered by chemical, physical, microbial, and antibody-antigen interactions (Ala’a et al., 2009). It is a local reaction and defensive mechanism that damages the live tissues of mammals and is designed to hinder the spread of harmful substances with the appearance of several symptoms, such as edema, leukocyte infiltration, and granuloma development (Amdekar et al., 2012).

Based on the duration and triggering stimuli, inflammation is classified into two types, acute and chronic (Ashour et al., 2018). The former is characterized by a rapid onset, brief duration, and migration of immune cells and antibodies from the bloodstream to the site of inflammation or injury (Morebise et al., 2002; Ayoub et al., 2021). It can be triggered by various factors, including bacterial, viral, fungal infections, toxins produced by microorganisms, hypoxia, ischemia, foreign bodies, tissue damage by chemical and physical injury, and immune-mediated responses. This response is typically protective, and is resolved once the foreign body is successfully removed. If the body cannot tackle the inflammatory reactions, acute inflammation may progress to chronic inflammation (Dinarello, 2010).

Wounds are physical damages that cause openings or disruptions in the skin, altering its normal assembly and function (Naz et al., 2013). They cause the epithelium to lose continuity, with or without essential loss of connective tissue and constitute a significant burden for both people and healthcare professionals worldwide, affecting approximately 6 million people worldwide (Nawaz et al., 2013). Untreated wounds continuously generate inflammatory mediators, causing discomfort and swelling at the wound site, which may result in death or multiple organ failure (Banik et al., 2006).

Wound-healing is a common biological consequence of an injury that undergoes a series of procedures. Although wound-healing is a well-known process, its biology is highly complex and is still only partially understood (Alqarawi et al., 2014). The wound-healing process occurs in three stages: inflammatory, proliferative, and remodeling. During the inflammatory phase, hemostatic systems are engaged to limit blood loss from the wound site, while the proliferative phase consists of three stages: granulation, contraction, and epithelialization. During granulation, fibroblasts form a collagen bed and fresh capillaries that aid in wound-healing (Adebiyi et al., 2018). During contraction, the wound edges help minimize gaps. In the third stage, new epithelial tissues are formed at the wound site. However, during renovation, new collagen is formed (Javed et al., 2019).

Natural products, particularly botanicals, constitute integral components of contemporary medicine when administered judiciously. Their significance lies in providing a potent, cost-effective therapeutic approach characterized by a lower incidence of side effects compared to pharmaceutical drugs. Notably, herbal medicine, assumes a critical role in the treatment and regulation of skin disorders, offering both high efficacy and minimal adverse effects. In developing nations, reliance on natural products for the management of skin conditions is commonplace. Moreover, their multifaceted advantages extend to antimicrobial, wound healing, and anti-inflammatory properties, offering a comprehensive spectrum of benefits in the treatment of diverse skin disorders (Mohd Zaid et al., 2022).

*Panicum antidotale* Retz is a tufted perennial grass that belongs to family Poaceae, and it grows up to 1.5–3 m tall (Franconie, 2000) and is primarily used as food and for grain production (Sebastin et al., 2018). The members of this family have wound healing activity (Jane et al., 2011). *Panicum antidotale* Retz contains various phytochemicals, such as flavonoids and phenols, which have been traditionally used for their antidiabetic, anxiolytic, analgesic, antibacterial, antioxidant, and anti-inflammatory properties. It has also been used as a poultice to alleviate local inflammation and painful diseases, such as arthralgia (Mustafa et al., 2020).

Limited information exists in the current literature regarding the metabolic profile and biological potential of *P. antidotale*. Therefore, this study aimed to investigate the *in vivo* anti-inflammatory and the wound-healing activities of its ethanol extract of the aerial parts (PAAPEE) for the first time. The former was performed using both acute models, such as histamine and carrageenan-induced paw edema in rates, as well as Freund’s complete adjuvant (FCA)-triggered arthritis as a chronic model, whereas the latter was investigated using an excision wound model. Additionally, *in vivo* analgesic and antipyretic activities were determined. Antioxidant potential was determined *in vitro* using a a 2,2-diphenyl-1-picrylhydrazyl (DPPH) free radical scavenging capacity assay. High performance Liquid Chromatography (HPLC) was used to identify and quantify the flavonoidal and phenolic contents of PAAPEE. Furthermore, the correlation between biological activity and the identified flavonoidal and phenolic metabolites was explored using molecular docking *versus* the active pockets of enzymes involved in the inflammation and wound-healing process, using Discovery Studio software.

## 2 Materials and methods

### 2.1 Chemicals and reagents

Various compounds were used in this investigation to develop anti-inflammatory and wound-healing models. In the acute models of *in vivo* anti-inflammatory experiments, carrageenan and histamine were utilized to induce inflammation. In the chronic models of *in vivo* anti-inflammatory examination, FCA was used. Meanwhile, DPPH was used in the antioxidant study.

### 2.2 Collection and extraction of plant material

The aerial parts of *P. antidotale* Retz (Poaceae) (3 kg) were collected (15 November 2020) from the National Park of Lal Sohanra, Bahawalpur, Pakistan and verified by Mr. Abdul Hameed, Botanist of the Cholistan Institute of Desert Studies, the Islamia University of Bahawalpur. The plant specimen was kept at the Department of Agriculture and Forestry, Bahawalpur, Pakistan and was allotted voucher no. 177. The whole plant was left to air-dry for 10 days before grinding. The plant material was subjected to three consecutive extractions with absolute ethanol (99%) until complete exhaustion (3 × 2 L). The extract was then filtered. The collected extract was dried using a rotary evaporator under vacuum conditions (Rotavapor R-200 Buchi, Germany) at 37°C, yielding 300 g of dried extract.

### 2.3 Experimental animals

Male *albino* rats purchased from Karachi, Pakistan, were used in the experiments. The animals were housed in a regulated environment with 50% relative humidity, 25°C temperature, and a 12 h light/dark cycle. They were provided with food and water regularly. The animals were acclimated to the laboratory conditions for 1 week before the start of pharmacological activities. The Animal Ethical Committee of Islamia University Bahawalpur, Pakistan authorized the research protocols and methods used in this study (AEC file no. PAEC/21/48).

### 2.4 Carrageenan-induced paw edema in rats

The animals were separated into five groups, each consisting of six rats. Group 1 was the negative control, in which the animals received 10 mg/kg of isotonic sodium chloride solution orally. Group 2 was the reference standard group, in which the animals received 20 mg/kg diclofenac sodium orally. In Groups 3–5, the animals were orally treated with 125, 250, and 500 mg/kg of PAAPEE, respectively. Subcutaneous injection of 0.1-mL of freshly prepared 2% w/v carrageenan was administered into the sub-plantar surface of each rat’s right hind paw after 1 h of pre-treatment. At 0, 1, 2, 3, 4, 5, and 6 h intervals, paw volume was quantified using a digital vernier caliper following carrageenan injection. The percentage of paw volume inhibition was determined using the following formula (Andleeb et al., 2022):

$$\%\;Inhibition=\frac{increase\;in\;paw\;volume\;\left(control\right)-increase\;in\;paw\;volume\;\left(test\right)}{Increase\;in\;paw\;volume\;\left(control\right)}\times 100$$

### 2.5 Histamine-induced paw edema in rats

The animals were separated into five groups, each consisting of six rats. Group 1 was the negative control, in which the animals were orally administered 10 mg/kg of isotonic sodium chloride solution. Group 2 was the reference standard group, in which the rats were orally pretreated with 20 mg/kg diclofenac sodium. However, in Groups 3–5, the animals were pretreated orally with 125, 250, or 500 mg/kg of PAAPEE, respectively. One hour after pretreatment, each rat in each group received 0.1 mL of freshly prepared histamine subcutaneously injected into the right hind paw subplantar surface. At 0, 1, 2, 3, 4, 5, and 6 h intervals, paw volume was quantified using a digital vernier caliper following carrageenan injection. The percentage of paw volume inhibition was determined using the following formula (Saleem et al., 2021a).

$$\%\;Inhibition=\frac{increase\;in\;paw\;volume\;\left(control\right)-increase\;in\;paw\;volume\;\left(test\right)}{Increase\;in\;paw\;volume\;\left(control\right)}\times 100$$

### 2.6 Chronic models for determination of anti-inflammatory activity using FCA-induced arthritis

This method is reliable for identifying persistent inflammation. FCA, which is *Mycobacterium tuberculosis* destroyed by heat and suspended in liquid paraffin, is used to induce arthritis (Gaspar et al., 2018). The animals were divided into five groups, each consisting of six rats. Group 1 was the negative control, in which the animals received 10 mg/kg of isotonic sodium chloride solution orally. Group 2 was the reference standard group, in which the rats were orally pretreated with 20 mg/kg diclofenac sodium. In Groups 3–5, the animals were pretreated orally with 125, 250, and 500 mg/kg of PAAPEE, respectively**.** A small amount 0.1 mL of 0.4% FCA was intradermally injected into the paw of the animals 24 h after administering test medications. Subsequently, the medication was continued daily for 21 days. The body weights and paw sizes of the rats were accurately measured. The percentage of arthritic inhibition was determined on days 1, 3, 5, 9, 13, and 21. Primary and secondary lesions are important criteria to assess arthritis. Initial lesions involve the development of edema in the injected paw, which reaches its peak 3–5 days after the stimulus from the inducing agent and is typically measured on the fifth day. Secondary lesions, such as inflammation in non-injected areas and weight loss in response to an immunological reaction, occur around 11–12 days after the injection (Saleem et al., 2021b).

### 2.7 Determination of analgesic activity

The analgesic activity of the mice was estimated using the writhing test, triggered by acetic acid, adopted by Koster et al. (1959). The animals were divided into five groups, each consisting of six rats: Group 1 was the control group, in which the animals received 20 mL/kg of isotonic sodium chloride solution orally. Group 2 was the reference standard group, in which the rats were orally administered 20 mg/kg diclofenac sodium. In Groups 3–5, the animals were administered 125, 250, or 500 mg/kg of PAAPEE, respectively. All the therapies were administered orally. After receiving the reference drug and test samples, each mice underwent an intraperitoneal injection of 10 mL/kg body weight of 0.7% acetic acid. The number of writhing reactions generated by each animal was counted for 15 min, starting 5 min after the injection of acetic acid. The following formula was used to determine the % of analgesic activity where W is the number of writhings, c is the control, and t is the test. (Amdekar and Singh, 2016).

$$\%\;Inhibition\;of\;abdominal\;writhing=\frac{\left(W_{C}-W_{t}\right)}{W_{C}}\times 100$$

### 2.8 Determination of antipyretic activity

The antipyretic effect was evaluated in rats using the usual method with slight modifications, utilizing the fever caused by Brewer’s yeast, where the rats became hyperthermic after being exposed to yeast. A digital thermometer was used to measure each rat’s basal rectal temperature at 0 h. Brewer’s yeast dissolved in distilled water (15% w/v) was subcutaneously administered at 10 mL/kg body weight to induce pyrexia. Animals with an increase in rectal temperature of at least 0.6°C (or 1°F) were chosen for the investigation. The increase in rectal temperature was measured 18 h after Brewer’s yeast injection. The rats were randomly divided into five groups of six animals each. Orally, Group 1 was administered with 1% isotonic sodium chloride solution, while Group 2 received the standard medicine, diclofenac sodium, at a peroral dose of 20 mg/kg. Groups 3–5 were orally administered 125, 250, and 500 mg/kg of PAAPEE, respectively. Following treatment, the body temperature of all animals in all groups was measured at 0, 1, 2, 3, and 4 h (Rakib et al., 2020; Abdelaty et al., 2021).

### 2.9 Determination of wound-healing activity using excision wound-healing model

Five groups, each with six animals, were used in the excision wound model: Group 1, which acted as the negative control and was treated with ointment base; Group 2, which represented the standard, received 20 mg of diclofenac sodium; and Groups 3–5, which were administered with 12.5, 25, and 50% (w/w) extract ointments, respectively. All the treatments were administered once daily.

To anesthetize the rats and produce an excision wound, ketamine hydrochloride (50 mg/kg body weight) was administered intraperitoneally and shaved with depilatory cream (Reckitt Benckiser, Inc., United Kingdom). An impression of the shaved dorsal area was taken to indicate the position of the incision to be performed. A metal punch was used to produce a 5-mm circular area along the marking. Naked rats were placed in an open environment. From the day of the procedure until full recovery, the basic ointment base, customized extract ointment, and regular medicine were administered once daily. The wound contraction and epithelialization time were measured using this model. Wound contraction was assessed as a percentage every fourth day after wound formation. All the rats were anesthetized at the end of the research. The edges of the healed wounds were left with a 5-mm margin of healthy skin, and tissue samples were collected from the healed wounds. Histopathological and biochemical tests were performed on specimen tissues maintained in a 10% formalin solution (Assaf et al., 2019; Yaseen et al., 2020).

### 2.10 *In vitro* DPPH assay

The proportion of antioxidant activity (AA %) of each item was determined using the DPPH free radical assay. Brand-Williams et al. established a method for measuring DPPH radical scavenging activity (11). The samples were treated with the stable DPPH radical in an ethanol solution. 0.5 mL sample, 3 mL absolute ethanol, and 0.3 mL 0.5 M DPPH radical solution in ethanol made up the reaction mixture. When DPPH interacts with an antioxidant molecule that can produce hydrogen, it is reduced. After 100 min of reaction, the colour changes (from deep violet to light yellow) were measured [Absorbance (Abs)] at 517 nm using a UV-VIS spectrophotometer (DU 800; Beckman Coulter, Fullerton, CA, United States). A mixture of ethanol (3.3 mL) and sample (0.5 mL) is employed as a blank. 3.5 mL ethanol was mixed with DPPH radical solution to make the control solution (0.3 mL). The proportion of scavenging activity (AA %) was determined (Greenberg et al., 1976).

$$AA\%=100-\lceil \frac{\left(Abs_{sample}-Abs_{blank}\right)x100}{Abs_{control}}\rceil$$

For each drug, the experiment was repeated three times. One-way ANOVA and Tukey’s test were used to compare the findings, which were reported as a % reduction compared to the control values. If *p* < 0.05, a difference was deemed statistically significant (Assaf et al., 2019).

### 2.11 HPLC analysis of flavonoids and phenolic metabolites in PAAPEE

HPLC was used to identify and quantify the flavonoid and phenolic contents of PAAPEE. The sample which is prepared by dissolving 10 mg of PAAPEE in 5 mL distilled water and 12 mL ethanol and after that the solution was vortexed to ensure homogeneity. The reaction mixture was then placed in an oven at 90°C for 2 h, after which distilled water (6 mL) was added, followed by 10 mL of 15 M of HCl. The reaction mixture was put in an oven at 90°C for 2 h (Sultana and Anwar, 2008). The samples were filtered using syringe filters. The flavonoids and phenolic metabolites were separated using a Shim-Pack CLC-ODS column (Shimadzu, Japan) where its stationary phase is octadecyl group; its particle size is 5 μm; its dimensions are 6.0 mm i. d X 15 cm with reversed phase as the separation mode and catalog number of 228-00808-91. The flow rate was 1 mL/min; the column length was 10 cm. Gradients A (H_2_O: AA-94:6, pH = 2.27), B (CAN 100%), 0–15 min = 15% V, 15–30 min = 45% B, and 30–45 min = 45% B were used in the mobile phase. A 280-nm UV-visible detector (SPD-10AV) and an LC-10AV pump were used (Hamed et al., 2021).

### 2.12 Molecular docking study

Virtual screening was done on HPLC-identified phenolic metabolites and flavonoids in the PAAPEE on 5-lipoxygenase (5-LOX) (PDB ID 3V99; 2.25 Å) and glycogen synthase kinase-3 *β* protein (GSK3*β*) (PDB ID 5K5N; 2.20 Å). These proteins were obtained from the protein data bank; docking was performed using Discovery Studio 4.5 (Accelrys Inc., San Diego, CA, United States) as per the C-Docker protocol (Labib et al., 2017; Thabet et al., 2018a; Talaat et al., 2018; Altyar et al., 2020), where binding energies (∆G) were assessed (Youssef et al., 2016).

### 2.13 Statistical analysis

Results are presented as mean ± standard deviation (SD). A *p*-value of 0.05 was considered significant when using the Student’s t-test to evaluate the statistical significance. GraphPad Prism (version 8.0.1) was used to compile and statistically analyze the data.

## 3 Results

### 3.1 Anti-inflammatory effects of PAAPEE in carrageenan-induced edema model

**FIGURE 1:**
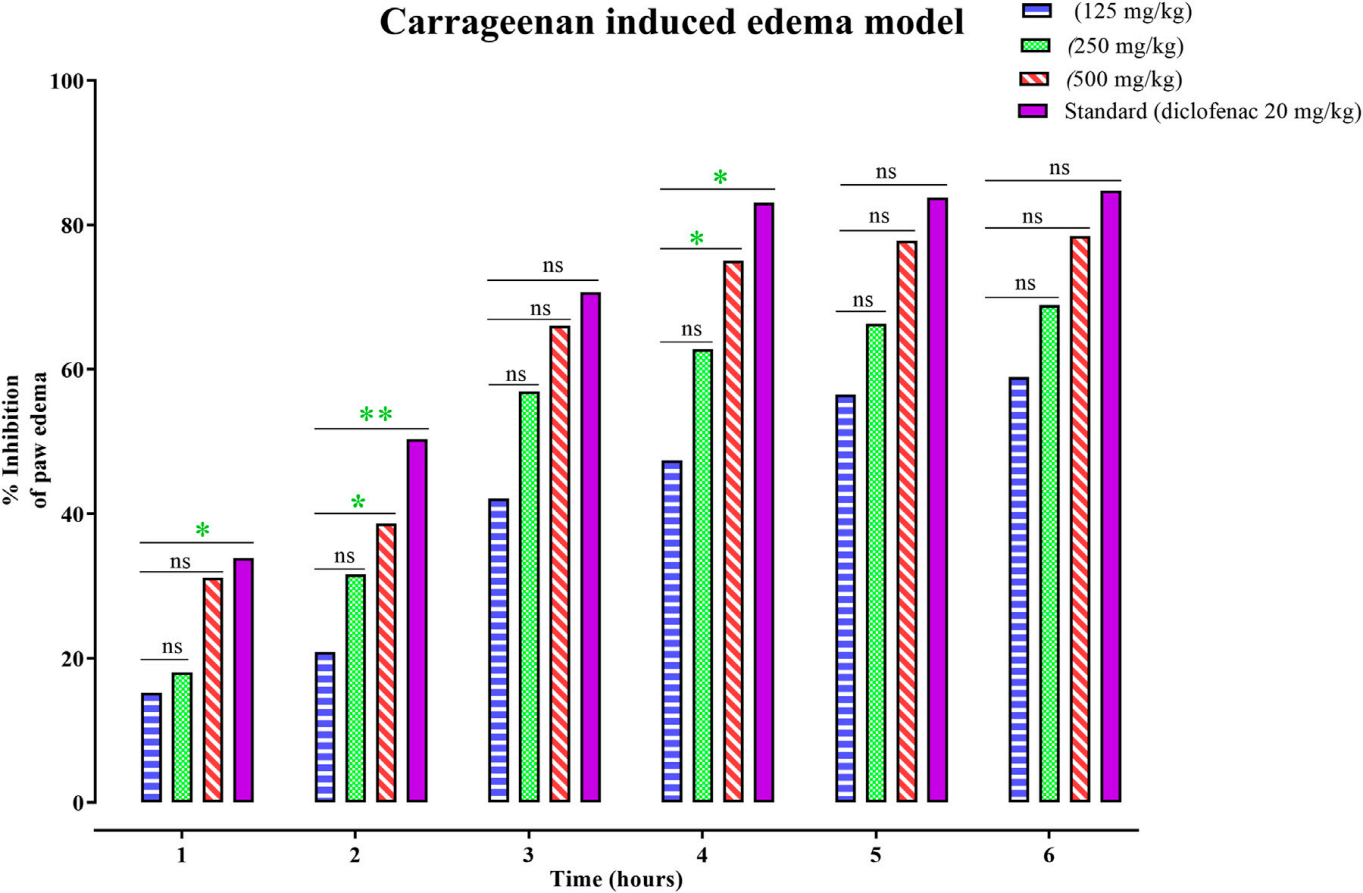
Effects 125, 250 and 500 mg/kg of PAAPEE on rat paw volume in carrageenan - induced edema model in comparison to standard (20 mg/kg of diclofenac sodium) during 6 h post treatment; where ns = non-significant, * = *p* < 0.05, ** = *p* < 0.01.

**FIGURE 2:**
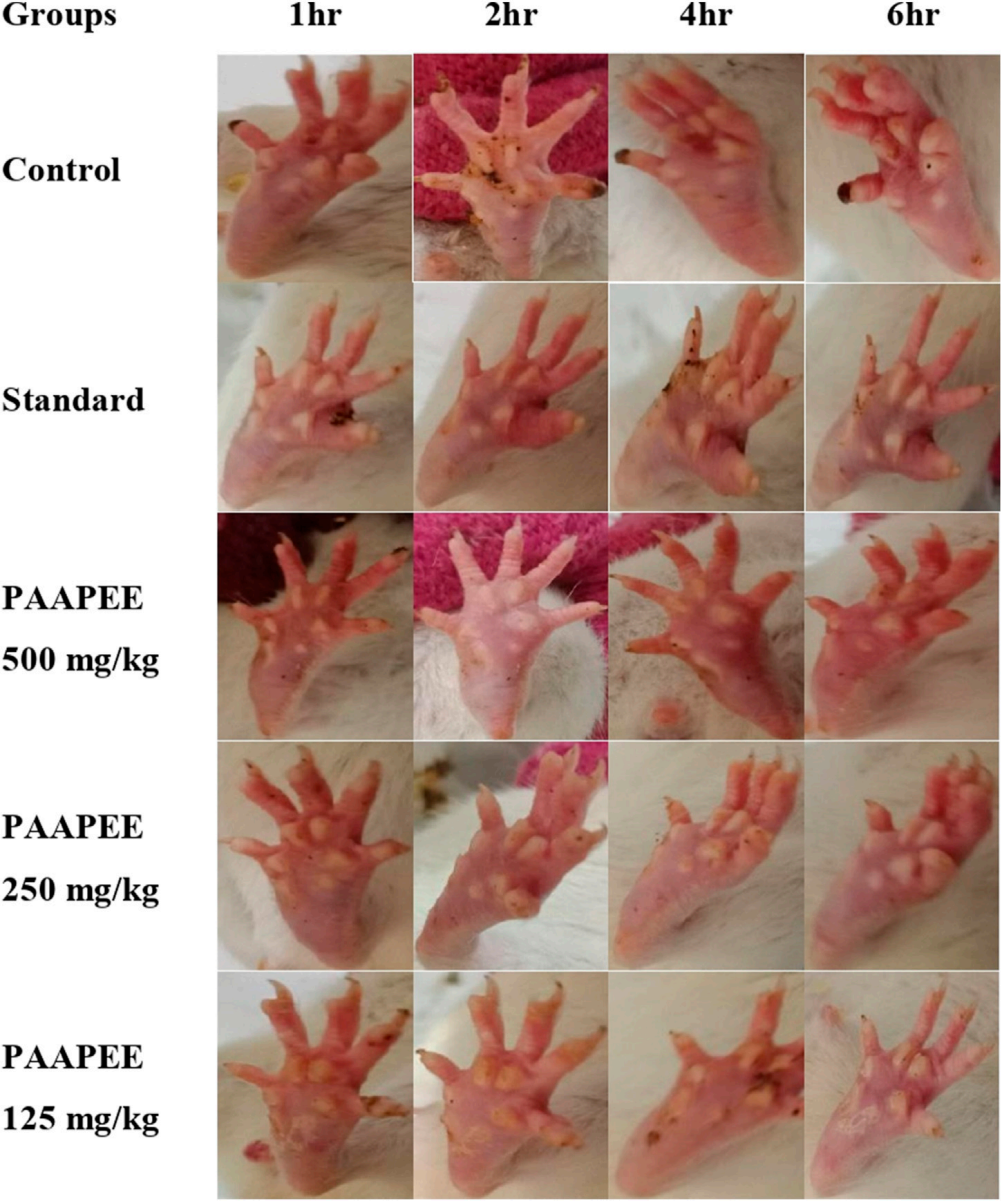
Macroscopic observation of paws in carrageenan-induced paw edema model during 6 h post treatment with 125, 250 and 500 mg/kg of PAAPEE and 20 mg/kg of diclofenac sodium.

The anti-inflammatory potential of PAAPEE using carrageenan-induced edema as an acute model was determined over 6 hours. The effects of PAAPEE on rat paw volume in a carrageenan-triggered edema model in comparison to the standard are illustrated in Figure 1. It was observed that the % inhibition (PI) of paw edema in groups that were administered 125, 250, and 500 mg/kg of PAAPEE and 20 mg/kg of diclofenac sodium was 15.21%, 18.03%, 31.15%, and 33.86%, respectively, compared to the diseased group that received isotonic sodium chloride solution only. However, the PI of paw edema in groups that were administered 125, 250, and 500 mg/kg of PAAPEE and 20 mg/kg of diclofenac sodium was 20.86%, 31.58%, 38.68%, and 53.06%, respectively, 2 h post-treatment; 42.09%, 56.95%, 66.06%, and 73.51%, respectively, 3 h post-treatment; 47.38%, 62.78%, 75.02%, and 86.09%, respectively, 4 h post-treatment; 56.52%, 66.35%, 77.78%, and 87.81%, respectively, 5 h post-treatment; and 58.92%, 68.85%, 78.44%, and 90.75%, respectively, 6 h post-treatment, when compared to that of the diseased group. It was clear that PAAPEE (500 mg/kg) displayed anti-inflammatory potential approaching that of diclofenac sodium in carrageenan-induced edema. Macroscopic observations of the paws in the carrageenan-triggered paw edema model are shown in Figure 2.

### 3.2 Anti-inflammatory effects of PAAPEE in histamine-induced edema

**FIGURE 3:**
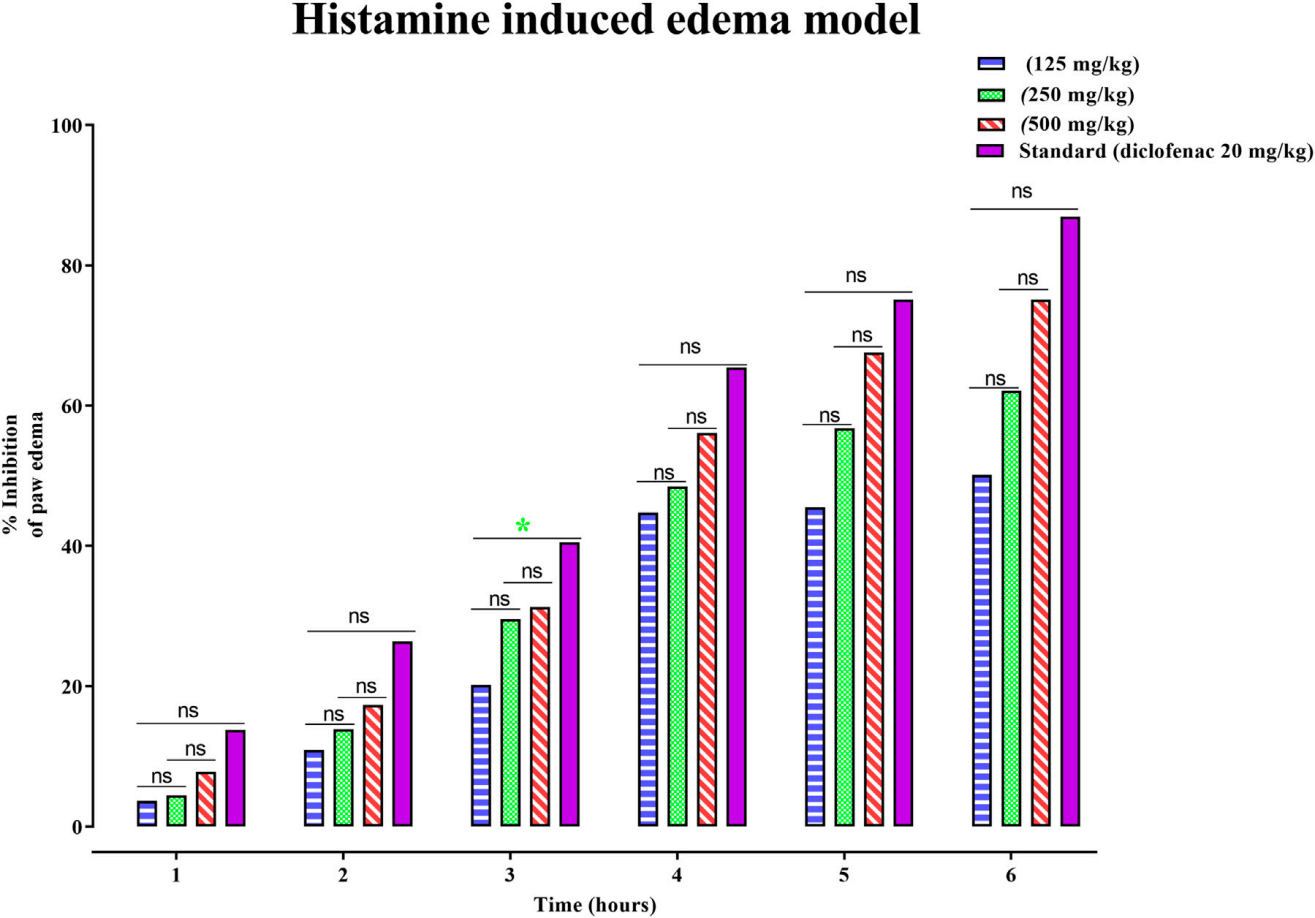
Effects 125, 250 and 500 mg/kg of PAAPEE on rat paw volume in histamine-induced edema model in comparison to standard (20 mg/kg of diclofenac sodium) during 6 h post treatment; where ns = non-significant, * = *p* < 0.05.

Similarly, the anti-inflammatory potential of PAAPEE in histamine-induced edema in an acute model was determined for 6 hours. The effects of PAAPEE on rat paw volume in a histamine-induced edema model in comparison to the standard are illustrated in Figure 3. It was observed that 1 h post-treatment, the PI of paw edema in groups that were administered 125, 250, and 500 mg/kg of PAAPEE and 20 mg/kg of diclofenac sodium was 3.68, 4.46, 7.80% and 13.80%, respectively, compared to that of the diseased group that received isotonic sodium chloride solution only. However, it was noted that the PI became 10.94%, 13.91%, 17.07%, and 26.40%, respectively, after 2 h of treatment; 20.15%, 29.53%, 31.35%, and 40.52%, 3 h post-treatment; 44.78%, 48.48%, 56.09%, and 65.46%, respectively, 4 h post-treatment; 45.51%, 56.78%, 67.59%, and 75.15%, respectively, 5 h post-treatment; and 50.14%, 62.17%, 75.13%, and 86.94%, respectively, after 6 h when compared to the untreated group. Thus, it is evident that PAAPEE, particularly at 500 mg/kg, showed an anti-inflammatory effect similar to that of diclofenac sodium in a histamine-triggered edema model.

### 3.3 Anti-inflammatory effects of *Panicum antidotale* in FCA-induced arthritis model

**FIGURE 4:**
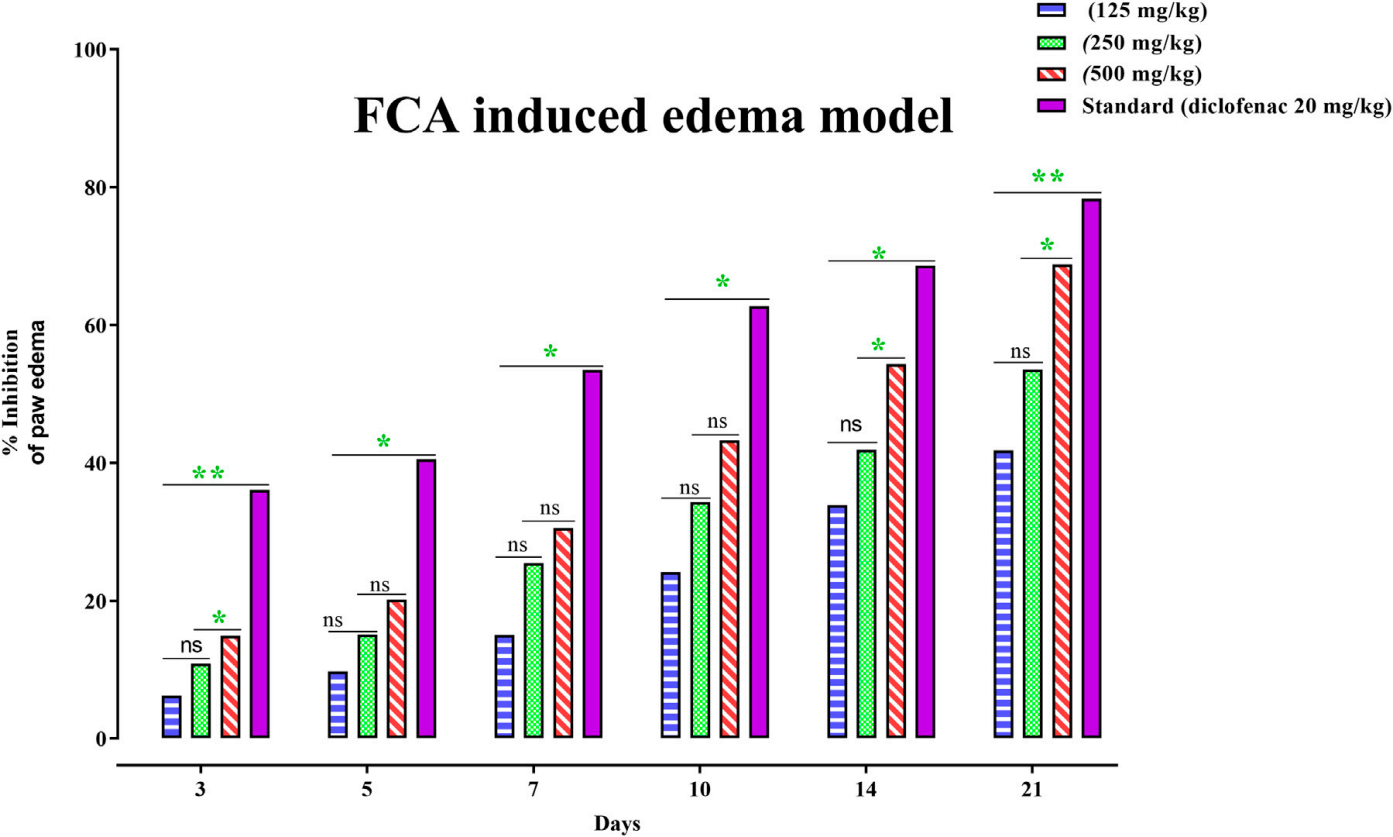
Effects 125, 250 and 500 mg/kg of PAAPEE on rat paw volume in FCA - induced edema model in comparison to standard (20 mg/kg of diclofenac sodium) during 6 h post treatment; where ns = non-significant, * = *p* < 0.05, ** = *p* < 0.01.

**FIGURE 5:**
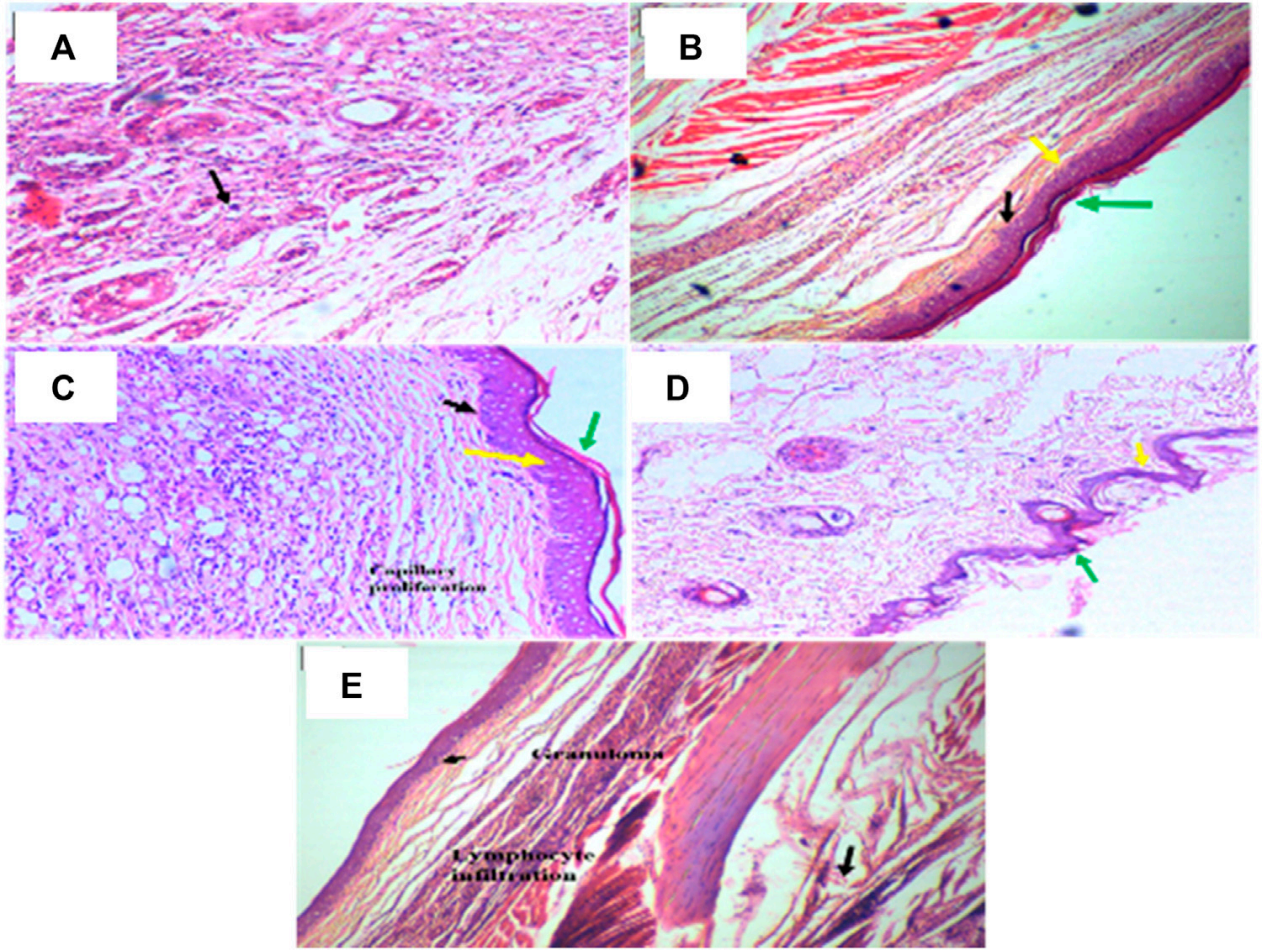
Histopathological examination of rat paws in FCA-induced edema model in diseased group **(A)**; Standard (diclofenac sodium) group **(B)**; PAAPEE treated groups with dosses 125 mg/kg **(C)**; 250 mg/kg **(D)**; and 500 mg/kg **(E)** The epidermal and dermal layers of the skin are represented by green and yellow arrows, respectively. In contrast, the underlying dermis shows localized fibroplasia (black arrow) and significant inflammation (red arrows) in all groups.

To alleviate chronic inflammation, the anti-inflammatory effects of PAAPEE were determined using FCA-triggered arthritis as a chronic inflammation model. The PI of paw edema was observed for 21 days after treatment with 125, 250, and 500 mg/kg of PAAPEE and 20 mg/kg of diclofenac sodium. On the third day post-treatment, the PI of paw edema in groups that were administered 125, 250, and 500 mg/kg of PAAPEE and 20 mg/kg of diclofenac sodium was found to be 6.23%, 10.83%, 14.89%, and 36.08%, respectively, compared to that of the untreated group. However, the PI of paw edema was notably elevated after treatment with 125, 250, and 500 mg/kg of PAAPEE and 20 mg/kg of diclofenac sodium to 9.74, 15.12, 20.14, and 40.48%, respectively, on the fifth day post-treatment; to 15, 25.46, 30.54, and 53.47%, respectively, on the seventh day post-treatment; to 24.13, 34.28, 43.22, and 62.73%, respectively, on the 10th day post-treatment; to 33.83, 41.88, 54.34, and 68.59%, respectively on the 14th day post-treatment and to 41.79, 53.50, 68.78, and 78.30%, respectively on the 21st day post-treatment compared to that of the untreated group (Figure 4). PAAPEE (500 mg/kg) significantly ameliorated inflammation, showing activity similar to that of diclofenac sodium in an FCA-induced arthritis model. Additionally, on the 22nd day, a histopathological examination of the FCA-induced paw edema model tissue was performed. The histopathological characteristics of the tissues from all animal groups are shown in Figure 5. Rat paw sections were examined microscopically using hematoxylin and eosin stain at ×10 magnification. It revealed significant inflammation and epithelial hyperplasia in the injured region of the paw in the control group (black arrow) (Figure 5A). The epidermal and dermal layers of the skin are represented by green and yellow arrows, respectively. In contrast, the underlying dermis shows localized fibroplasia (black arrow) and significant inflammation (red arrow) in all groups. Minimal cutaneous inflammation was detected in the injured region of the paw in the diclofenac sodium (20 mg/kg)-treated group (Figure 5D). Lymphocytic infiltration and granulomas were observed in the injured area of the paw in the (500 mg/kg) treated animals, and inflammation is represented by black arrows (Figure 5E).

### 3.4 Analgesic effect of PAAPEE in acetic acid-induced pain model

**FIGURE 6:**
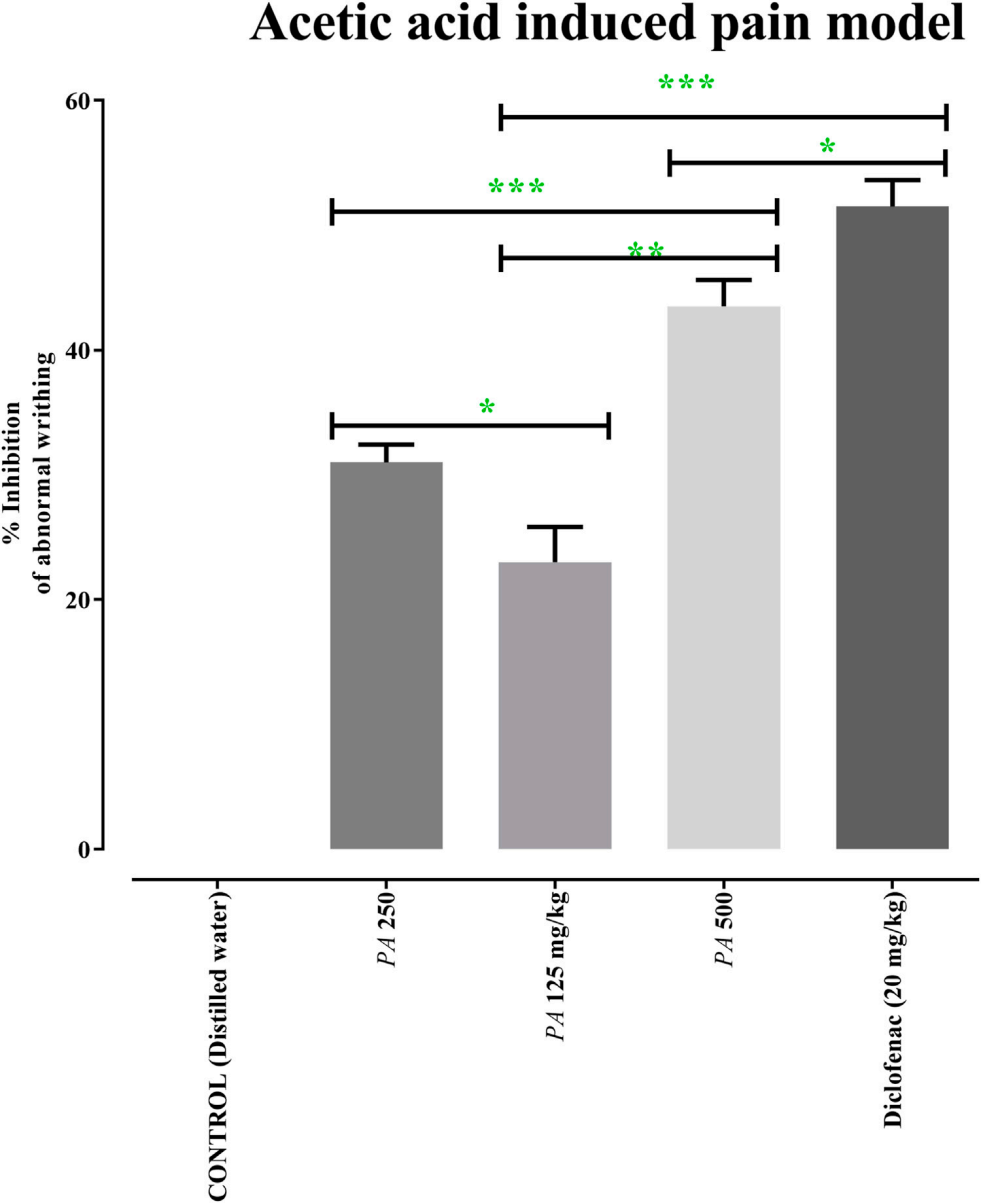
Effects of 125 (PA-125), 250 (PA-250) and 500 (PA-500) mg/kg of PAAPEE on acetic acid induced pain model in comparison to standard (20 mg/kg of diclofenac sodium) and control that received distilled water*;* where ns = non-significant, * = *p* < 0.05, ** = *p* < 0.01, *** = *p* < 0.001.

After acetic acid administration, the number of writhing events was calculated in the first 30 min for 15 min. Administration of 125, 250, and 500 mg/kg of PAAPEE and 20 mg/kg of diclofenac sodium showed analgesic effects evidenced by PI of abnormal writhing estimated at 23%, 31%, 44%, and 51%, respectively, compared to that of the untreated group 30 min post-treatment (Figure 6).

### 3.5 Antipyretic effect of PAAPEE in yeast-induced fever model

**FIGURE 7:**
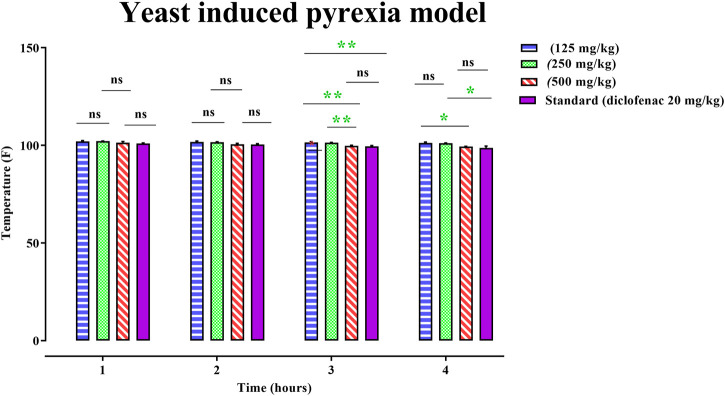
Effects 125, 250 and 500 mg/kg of PAAPEE on yeast induced pyrexia model in comparison to standard (20 mg/kg of diclofenac sodium) during 4 h post treatment; where ns = non-significant, * = *p* < 0.05, ** = *p* < 0.01.

The rats’ rectal temperature was measured at 1, 2, 3, 4, and 18 h after yeast administration to the animal model, and the mean animal temperature was recorded. By examining the animal’s temperature, the administration of 125, 250, and 500 mg/kg of PAAPEE and 20 mg/kg of diclofenac sodium showed a pronounced decline to the elevated body temperature of 102, 102.15, 101.4, and 101.05°F, respectively, during the first h post-treatment; 101.75, 101.7, 100.55, and 100.4°F, respectively, during the second h post-treatment; 101.55, 101.45, 99.85, and 99.5°F, respectively, during the third h post-treatment; and 101.3, 101.1, 99.45, and 98.8°F, during the fourth h post-treatment. Hence, PAAPEE at a dose of 500 mg/kg revealed nearly similar antipyretic potential as diclofenac sodium (Figure 7).

### 3.6 Wound-healing activity of PAAPEE using the excision healing of wound model

**FIGURE 8:**
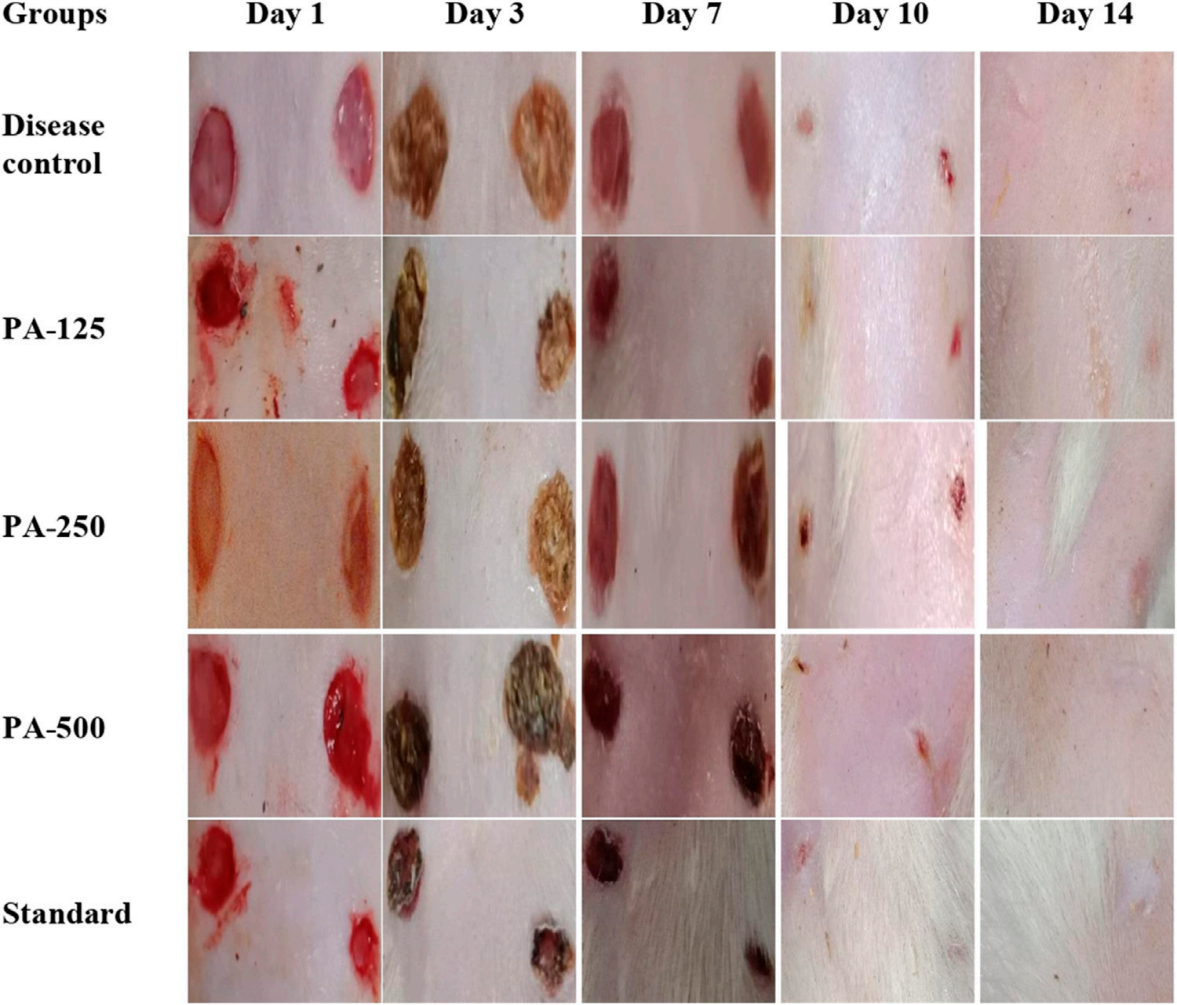
Macroscopic observation of skin in wound-healing activity for 125 (PA-125), 250 (PA-250) and 500 (PA-500) mg/kg of PAAPEE in comparison to standard (20 mg/kg of diclofenac sodium) and control.

**FIGURE 9:**
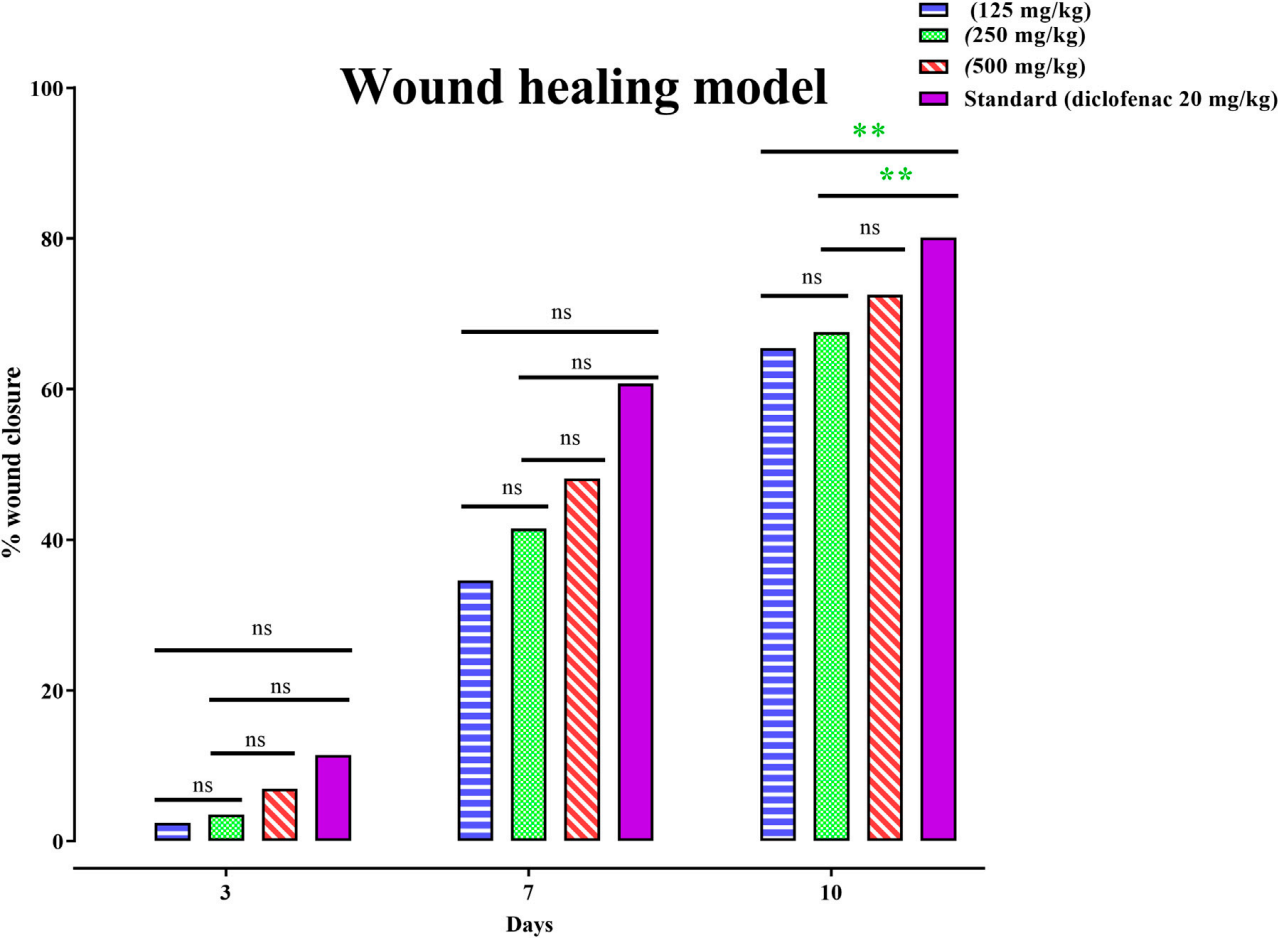
Effects 125, 250 and 500 mg/kg of PAAPEE on healing of wound model in comparison to standard (20 mg/kg of diclofenac sodium) during 4 h post treatment; where ns = non-significant, * = *p* < 0.05, ** = *p* < 0.01.

**FIGURE 10:**
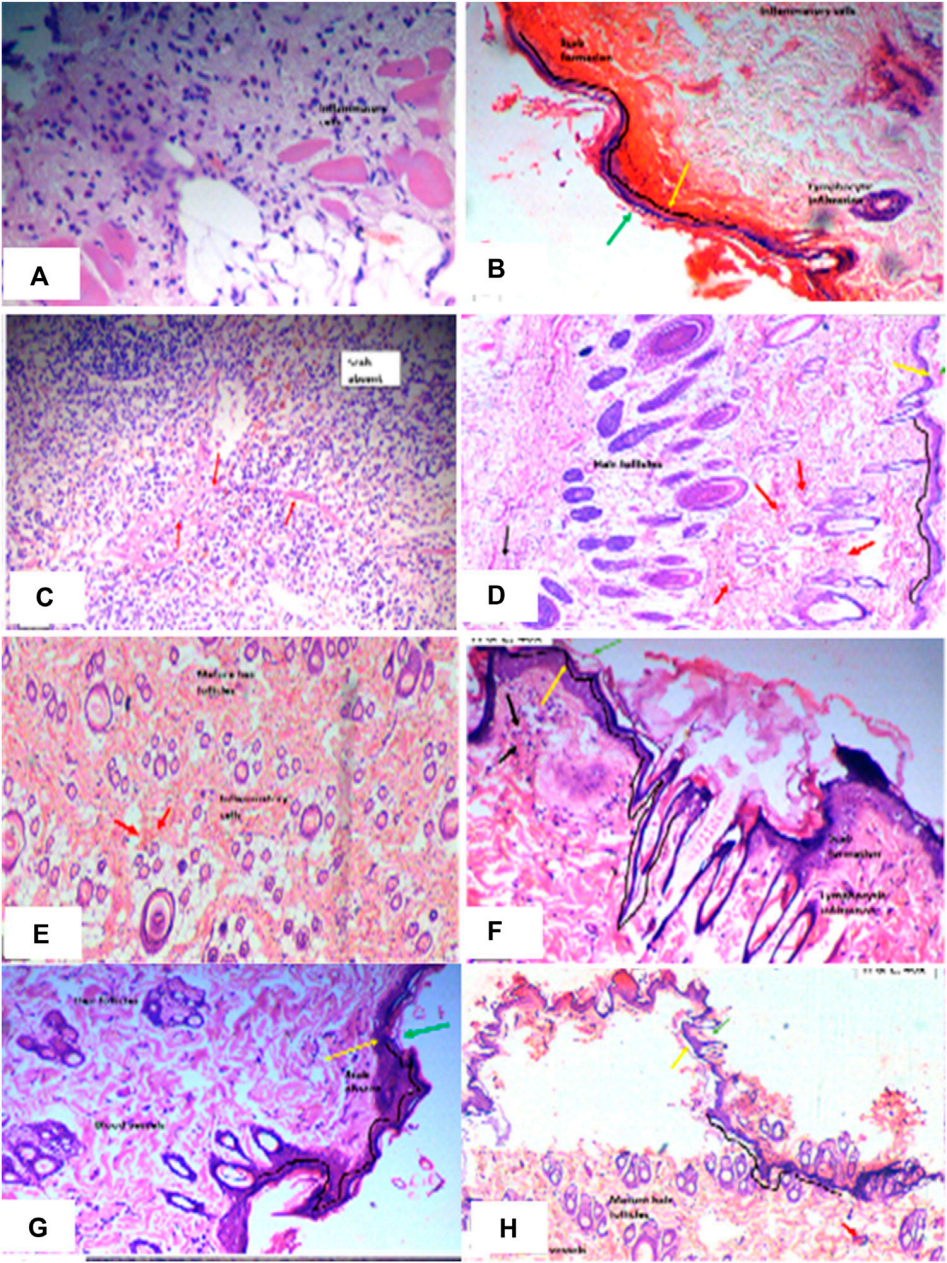
Histopathological evaluation of skin samples of wound-healing activity in diseased group **(A)**; Standard (diclofenac sodium) group **(B, C)**; PAAPEE treated groups with dosses 125 mg/kg **(D, E)**; 250 mg/kg **(F, G)**; and 500 mg/kg **(H)** The epidermal and dermal layers of the skin are represented by green and yellow arrows, respectively. In contrast, the underlying dermis shows localized fibroplasia (black arrow) and significant inflammation (red arrows) in all groups.

Wound-healing activity of PAAPEE was determined on the 10th day post-treatment where administration of 125, 250, and 500 mg/kg of PAAPEE and 20 mg/kg of diclofenac sodium revealed wound-healing potential evidenced by % wound closure of 2.44%, 3.55%, 6.98%, and 11.44%, respectively, compared to that of the untreated group on the third day post-treatment. In contrast, they showed enhancement in wound closure by 34.60%, 41.47%, 48.11%, and 60.73%, respectively with respect to that of the untreated group on the seventh day post-treatment and 65.45%, 67.57%, 72.52% and 80.12%, respectively, on the 10th day post-treatment. On the 14th day post-treatment, complete wound-healing was observed in all groups (Figures 8, 9). Comparison between 500 mg/kg of PAAPEE and 20 mg/kg diclofenac sodium groups showed substantial activity with a non-significant difference noted between them. On the 12th day, the wound tissues were histologically evaluated with hematoxylin and eosin staining. The histopathological characteristics of tissues illustrated in Table 1 and Figure 10 showed that reduced collagen fibers, blood vessels, fibroblast cells, inflammatory cells, and evident scar tissue were detected in a slice of Group 1 mice (control) (Figure 10A). However, full tissue regeneration was shown in Group 2 (standard), as evidenced by increased collagen fibers, fibroblast cells, and blood vessels, in addition to reduced inflammatory cells (Figures 10B, C). In contrast, Group 3 (12.5% w/w ointment) displayed less cellular necrosis and more blood vessels and collagen fibers (Figures 10D, E). Group 4 showed improved fibroblast and blood vessel growth (25% w/w/ointment) (Figures 10F, G). Group 5 (50% w/w ointment) had considerably well-organized collagen fibers with more fibroblast cells and blood vessels (Figure 10H) than that in the control. In all groups, complete re-epithelization (black line) was seen, whereas scab development was seen in the control, 25, and 50% of PAAPEE groups. In 50% of PAAPEE treated groups, granulation was detected but was absent in all other groups. The epidermal and dermal layers of the skin are represented by green and yellow arrows, respectively. In contrast, the underlying dermis shows localized fibroplasia (black arrow) and significant inflammation (red arrow) in all groups. Inflammation and blood cells were detected in all groups.

**TABLE 1:** Histopathological evaluations of skin in wound-healing activity of 125 (PA-125), 250 (PA-250) and 500 (PA-500) mg/kg of PAAPEE in comparison to standard (20 mg/kg of diclofenac sodium) and control.

	Control	Standard	PA-125	PA-250	PA-500
Scab formation	N/L	++	-	+	+
Complete re-epithelization	+	+	+	+	+
Fibroblast of Sub-epidermal tissues	++	++	+	++	++
Granulation tissues	-	-	-	N/L	+
Blood vessels	+	+	+	+	+
Inflammatory cell types	+	+	+	+	+
Intensity of inflammation	Mild	Mild	Mild	Mild	Mild
Epidermal changes	Thin	Thick	Thin	Thick	Thin

### 3.7 *In vitro* antioxidant activities of *Panicum antidotale using* DPPH

Results of *in vitro* antioxidant assays of *P. antidotale* in contrast with standard revealed that it showed IC_50_ of 62.50 ± 6.85 μg/mL in contrast to standard, ascorbic acid, that showed IC_50_ of 85.51 ± 0.38 μg/mL; Values shown are mean ± SD of three independent experiments.

### 3.8 HPLC analysis of flavonoids and phenolic metabolites in PAAPEE

**FIGURE 11:**
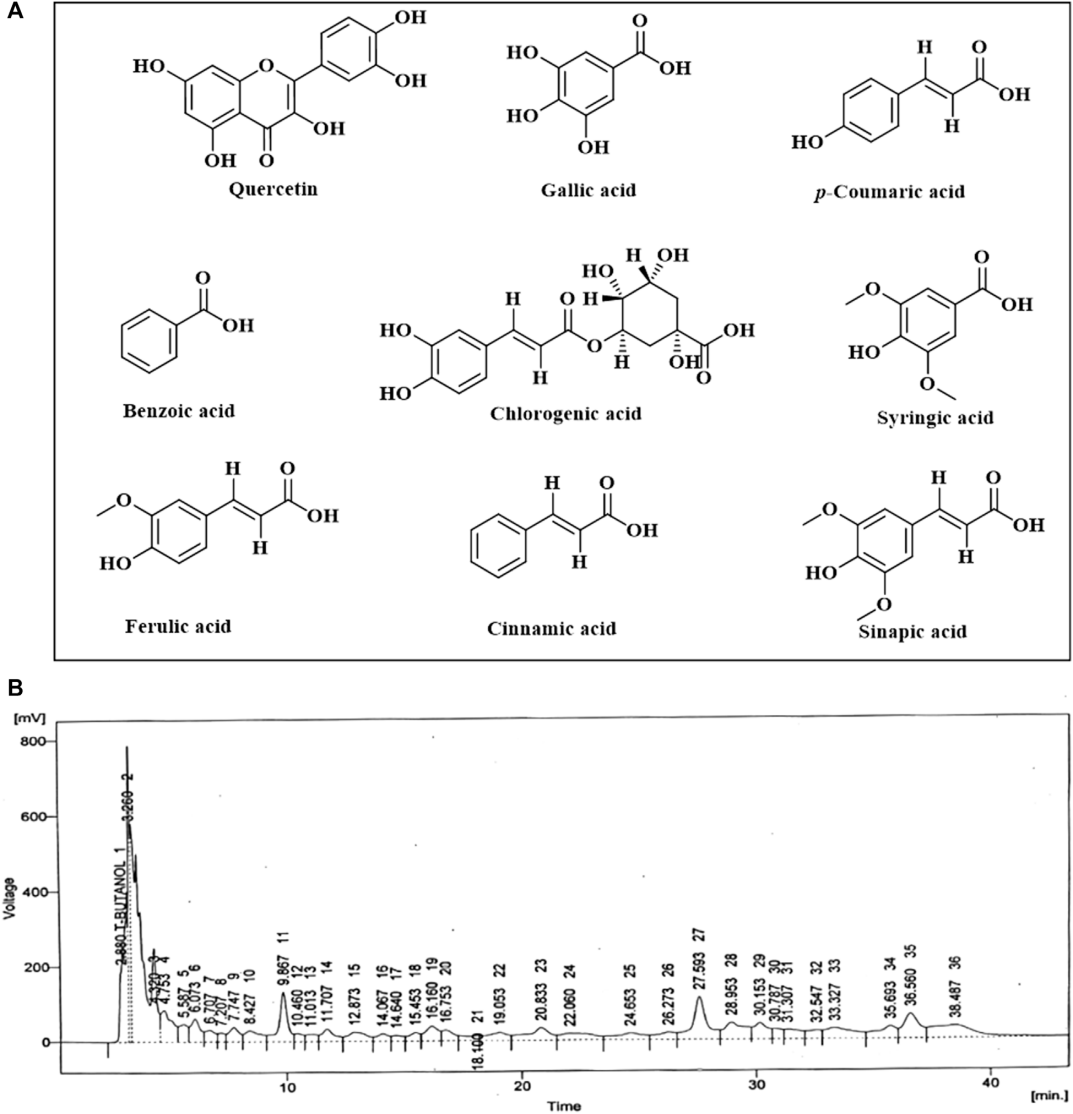
A scheme showing the identified flavonoids and phenolic metabolites in PAAPEE by HPLC analysis **(A)**; HPLC chromatogram **(B)**.

HPLC analysis of phenolic metabolites and flavonoids in PAAPEE (Table 2) showed the existence of quercetin, gallic acid, *p*-coumaric acid, benzoic acid, chlorogenic acid, syringic acid, ferulic acid, cinnamic acid, and sinapic acid. A schematic representation of the identified metabolites is shown in Figures 11A, B.

**TABLE 2:** The identified flavonoids and phenolic metabolites in PAAPEE by HPLC analysis.

Compounds	Synonym	Molecular weight	Retention time	Molecular formula	UV *λ*max (nm)	Area (%)
Benzoic acid	• Benzoic acid	122.12 g/mol	14.640	C_6_H_5_COOH	227	0.6
	• Dracylic acid					
	• Benzenecarboxylic acid					
	• Carboxybenzene					
Chlorogenic acid	• 1,3,4,5-tetrahydroxycyclohexanecarboxylic acid 3-(3,4-dihydroxycinnamate)	354.31 g/mol	15.453	C_16_H_18_O_9_	325	0.9
	• 3-Caffeoylquinic acid					
	• 3-(3,4 dihydroxycinnamoyl) Quinic acid					
Cinnamic acid	• *Z* (cis)-Cinnamic acid	148.16 g/mol	24.653	C_9_H_8_O_2_	273	1.8
	• *E* (trans)-Cinnamic acid					
Coumaric acid	• p-Coumaric acid	164.16 g/mol	12.873	C_9_H_8_O_3_	286	1.8
	• 4-Hydroxycinnamic acid					
	• p-Hydroxycinnamic acid					
	• trans-4-Hydroxycinnamic acid					
Ferulic acid	• trans-Ferulic Acid	194.18 g/mol	22.060	C1_0_H_10_O_4_	316	2.2
	• 4-Hydroxy-3-methoxycinnamic acid					
Gallic acid	• 3,4,5 Trihydroxybenzoic Acid	170.12 g/mol	4.753	C_7_H_6_O_5_	272.5	3.8
Quercitin	• Meletin	302.24 g/mol	3.260	C_15_H_10_O_7_	256	6.0
	• Sophoretin					
	• Quercetine					
Sinapic acid	• 4-Hydroxy-3,5-dimethoxycinnamic acid	224.21 g/mol	26.273	C_11_H_12_O_5_	323	1.3
	• 3-(4-hydroxy-3,5-dimethoxyphenyl)prop-2-enoic acid					
Synergic acid	• 4-Hydroxy-3,5-dimethoxybenzoic acid	198.17 g/mol	16.753	C_9_H_10_O_5_	216	1.3
	• 3,5-Dimethoxy-4-hydroxybenzoic acid					
	• Cedar acid					

### 3.9 Molecular docking study

**FIGURE12:**
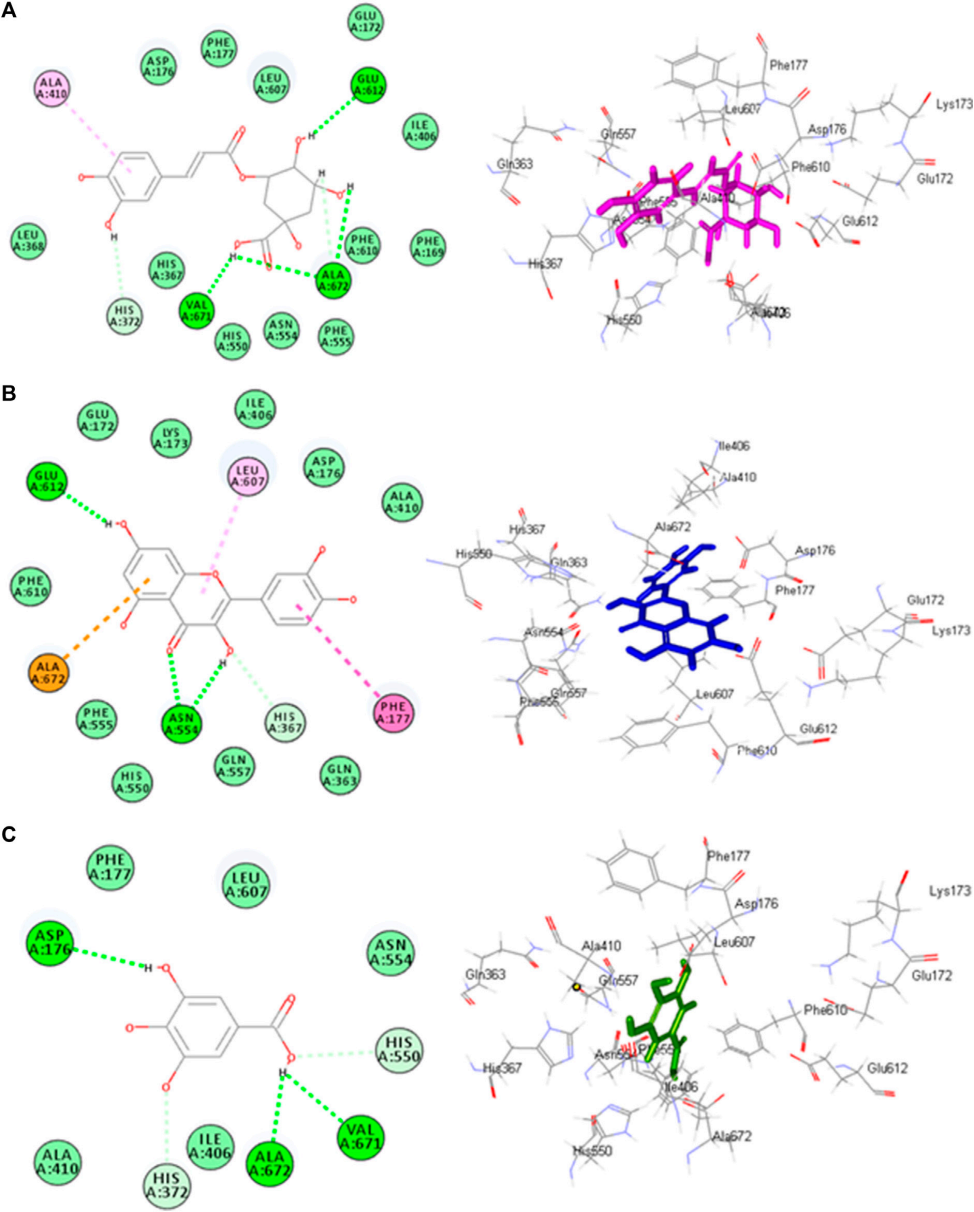
2D and 3D binding modes chlorogenic acid **(A)**, quercitin **(B)** and gallic acid **(C)** within the active sites of 5-LOX.

**FIGURE13:**
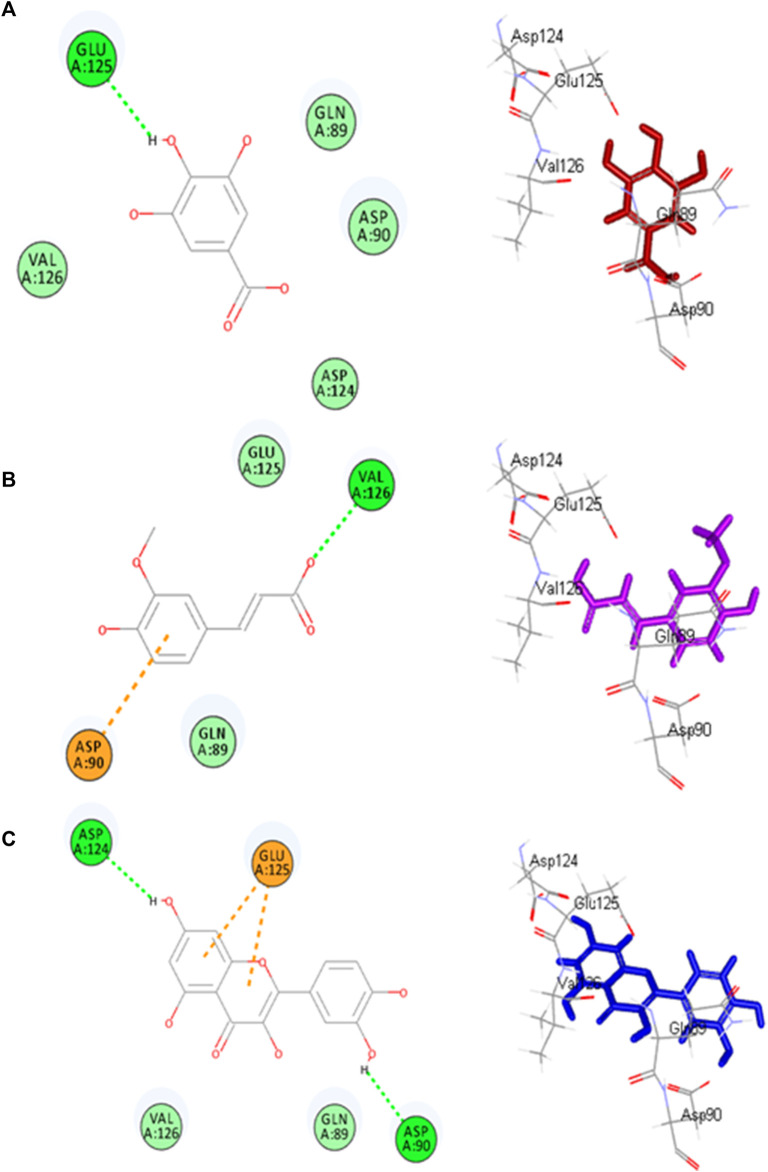
2D and 3D binding modes gallic acid **(A)**, ferulic acid **(B)** and quercitin **(C)** and within the active sites of GSK3-*β*.

Molecular docking investigations were done on HPLC-identified phenolic metabolites and flavonoids in PAAPEE on 5-LOX (incorporated in the inflammatory process) and GSK3*β* (incorporated in the process of wound-healing). Table 3 showed that all the tested metabolites revealed notable activity within the tested enzyme active sites; however, chlorogenic acid, quercetin, and gallic acid displayed the best fitting within the active sites of 5-LOX with free binding energy (∆G) values of −35.39, −34.70, and −32.44 kcal/mol, respectively approaching that of nordihydroguaiaretic acid (NDGA) (5-LOX Co-crystalized ligand) and potent 5-LOX inhibitor (∆G = .-43.37 kcal/mol). Meanwhile, gallic acid, ferulic acid, and quercetin exerted the best fitting within the GSK3*β* binding center with (∆G) values of −26.55, −19.73, and −19.46 kcal/mol, respectively showing superior activity when compared to PF-04802367 (PF-367) which is a highly selective GSK-3 inhibitor and it is co-crystalized with the protein (∆G = .-12.10 kcal/mol). From Figure 12A, it is clear that chlorogenic acid forms many notable interactions with the amino acids of the 5-LOX active pocket, manifested by four H-bonds with Glu612, Ala672, and Val671; one *π*-alkyl bond with Ala410; and two C-H bonds with Ala672 and His327. Furthermore, quercetin forms three H-bonds with Glu612, Asn554; one *π*-anion bond with Ala672; *π*-π bond with Phe177; *π*-alkyl bond with Leu607 together with one C-H bond with His367 (Figure 12B) whereas gallic acid forms three H-bonds with Asp176, Ala672, Val671; and two C-H bonds with His372, His550 (Figure 12C). Regarding GSK3*β* binding center, Figure 13A showed that gallic acid forms one H-bond with Glu125; ferulic acid showed one H-bond interaction with Val126; one *π*-anion bond with Asp90 (Figure 13B); however, quercetin forms two H-bonds with Asp124, Asp90, and two *π*-anion bonds with Glu125 (Figure 13C).

**TABLE 3:** Free binding energies (kcal/mol) of the identified flavonoids and phenolic metabolites in PAAPEE by HPLC analysis using *in silico* studies within 5-lipoxygenase (5-LOX) and glycogen synthase kinase3-*β* protein binding sites (GSK-3 *β*).

Compound	(5-LOX)	Number of formed bonds	(GSK-3 *β*)	Number of formed bonds
Benzoic acid	−15.84	-	−14.60	2; Lys91, Asp90
Chlorogenic acid	−35.39	7; Glu612, Ala672, Ala410, Val671, His327	−16.59	6; Glu89, Asp90, Leu88, Lys94
Cinamic acid	−21.84	4; Asn554, Ala672, Val671, His327	−12.79	2; Lys91, Asn95
Ferulic acid	−28.88	6; Val671, His550, Asn554, Asp176, Leu607	−19.73	2; Asp90, Val126
Gallic acid	−32.44	5; Asp176, His372, Ala672, Val671, His550	−26.55	1; Glu125
Coumaric acid	−27.95	4; Ala672, Val671, His550, Asn554	−16.21	2; Lys91, Asp90
Quercitin	−34.70	7; Glu612, Ala672, Asn554, Phe177, Leu607, His367	−19.46	4; Asp124, Asp90, Glu125
Sinapic acid	−27.19	9; Asp176, Ala672, Val671, His550, Asn554, Leu607, His367, Gln557	−16.76	3; Asp90, Val126
Synergic acid	−23.41	5; Asp176, Ala672, Asn554, His367, Gln557	−15.06	1; Glu125
Nordihydroguaiaretic acid (NDGA) (5-LOX Co-crystalized ligand)	−43.37	9; Pro569, Arg569, His360, His432, Phe359, Ala410, Leu607, His367	-	ND
PF-367 (GSK-3 *β* co-crystalized ligand)	-	ND	−12.10	10; Val135, Val70, Lys85, Ala83, Leu132, Leu188, Val110, Asp133

ND, not done; PF-04802367 (PF-367) is a highly selective GSK-3 inhibitor and it is co-crystalized with the protein.

## 4 Discussion

Inflammation is an acute component of the protective mechanisms of the immune system against invading pathogens. The response is characterized by redness, swelling, discomfort, and edema (Sheeja et al., 2006). Different microorganisms and endogenous and external stimuli cause a cascade of events that ultimately result in inflammation (Mutahar et al., 2012). The acute inflammatory response is the first reaction of the body *versus* an invading agent triggered by the immune system. It has a quick onset and brief duration, and moves immune cells from the blood circulation to the site of inflammation or injury, in contrast to chronic inflammation, which is characterized by a long reaction of the body to injury or inflammation (Panossian et al., 2000). If the body cannot deal with an inflammatory reaction, it will progress to chronic inflammation and become chronic inflammation. Chronic inflammation is exacerbated by reactive oxygen species. They can persist from a few days to several months. In this type of inflammatory response, tissue damage and healing occur simultaneously but at distinct rates (Rujjanawate et al., 2003). Several illnesses, such as arthritis, diabetes, cancer, and autoimmune diseases, may be exacerbated by chronic inflammation, including the common cold (Ullah et al., 2014a).

Using non-steroidal anti-inflammatory drugs (NSAIDs) for an extended period increases the risk of developing gastrointestinal ulcers, bleeding difficulties, and renal problems. Consequently, medications or cures must be developed to benefit patients suffering from chronic and acute illnesses while also having a more favorable therapeutic profile and a lower incidence of adverse effects (Meera et al., 2018). This study tested the anti-inflammatory, antipyretic, analgesic, and wound-healing potential of PAAPEE as a natural plant product at various concentrations. It contains various phytochemicals, including flavonoids and phenols, traditionally used for their antidiabetic, anxiolytic, analgesic, antibacterial, antioxidant, and anti-inflammatory (Mustafa et al., 2020). It has also been employed as a poultice for local inflammation and alleviation of painful diseases, such as arthralgia (Godugu et al., 2018).

Meanwhile, inflammatory responses are caused by several mechanisms, such as carrageenan-induced paw edema, which is divided into two stages: the first stage (mainly reliant on histamine release, bradykinin, and serotonin and lasting between one and 2 hours) and the second stage (lasting between three and 4 hours, mainly reliant on the release of bradykinin and lysozyme). The results showed that nitric oxide is crucial for controlling the permeability of blood vessels and stimulating cell migration induced by pro-inflammatory chemicals, such as carrageenan (Hossain et al., 2013). NSAIDs may aid in preventing edema, particularly in the late stages of the disease when prostanoid derivatives are present (Takaki et al., 2008). PAAPEE at 125, 250, and 500 mg/kg substantially decreased carrageenan-induced paw edema in mice. After 3 h of carrageenan administration, PAAPEE particularly at 500 mg/kg, showed higher anti-inflammatory efficacy than 250 mg/kg.

Histamine is a powerful vasodilator and an inflammatory mediator in the body; it also boosts vascular permeability (Vasudevan et al., 2006). Histamine increases the production of macrophage-1 antigen and Intercellular Adhesion Molecule 1 (ICAM-1) in eosinophils, allowing them to migrate more easily to the site of inflammation and exacerbate it. When histamine binds to H4 receptors on mast cells, calcium is released, prompting the mast cells to move to the inflammatory site. When histamine attaches to H1 receptors in pulmonary macrophages, it generates IL-6, cytokines, and glucuronidase, all of which are harmful to the body. When histamine binds to H2 receptors on the surface of peritoneal macrophages, it suppresses the production of Tumor necrosis factor-alpha (TNF-α), and IL12. Because of its stimulatory effect on NF-KB, it promotes the synthesis of inflammatory mediators and prostaglandins (La Manno et al., 2018). In a histamine-induced paw edema model, PAAPEE at 125, 250, and 500 mg/kg substantially decreased the progression of paw edema. After 2 h, the 500 mg/kg PAAPEE exhibited higher anti-inflammatory efficacy than that of the 250 mg/kg PAAPEE. Because of its capacity to inhibit the production of prostaglandins and other histamine-like mediators that cause edema, PAAPEE may have anti-inflammatory effects.

A chronic inflammatory condition known as FCA-induced arthritis presents in two stages: an acute phase (beginning on day 0), in which inflammatory reactions occur locally, resulting in edema in the paw that is injected with FCA as primary arthritic lesions, and a chronic inflammatory phase (beginning on day 15), manifested by edema that develops in the opposite paw as secondary arthritic lesions. Leukocytes migrate to damaged areas and generate edema, producing histamine, the cytokines IL-1 and TNF-α, serotonin, kinins, and prostaglandins during the first acute phase. IL-6 is the major mediator in the degeneration of bone and cartilage (Hua et al., 2005). The anti-arthritic effects of drugs can be assessed using a variety of parameters, including arthritis score and hind paw volume. The inflammatory response after FCA immunization in the acute phase was determined by assessing the primary arthritic lesion in the injected hind paw. In contrast, the generalized immune response was determined by evaluating the secondary arthritic lesion in the control hind paw, which leads to polyarthritis in the chronic phase (Akhtar and Shabbir, 2019). The animal groups given diclofenac sodium 20 mg/kg or 500 mg/kg PAAPEE displayed a notable decrease in the volume of the injected hind paw compared to the arthritic FCA group. This finding indicates that 500 mg/kg PAAPEE reduced inflammation during the acute stage. It also demonstrated a strong 99% inhibitory effect on inflammation and significantly decreased the volume of the contralateral paw during the subsequent phase of inflammation.

When acetic acid is injected intraperitoneally, the characteristic pain activity is abdominal muscle contraction, accompanied by hind limb extension and lengthening of the affected body part (Park et al., 2009). The local peritoneal receptors mediate this constriction. A quick and popular method for testing analgesic drugs is writhing in response to acetic acid. Acetic acid administration releases endogenous chemicals, which, in turn, stimulate the nerve terminals responsible for pain. Peritoneal fluid treated with allogeneic acetic acid showed elevated levels of prostaglandins, specifically PGE2, PGF2, PGI2, peritoneal mast cells, and lipoxygenase products. With an increase in capillary permeability, acetic acid intensifies the pain. The interaction of prostaglandins with endogenous mediators, including substance P, histamine, bradykinin, and serotonin, which further increases the sensitivity of pain receptors to these mediators, is the principal mechanism by which prostaglandins elicit a pain response. NSAIDs reduce pain by preventing the synthesis of prostaglandins, thromboxanes, and other inflammatory mediators via the action of cyclooxygenases. All analgesic drugs reduce the amount of constriction caused by acetic acid is lessened by all analgesic drugs (Ilmi et al., 2021). In a dose-dependent manner, the PAAPEE dramatically decreased the number of writhing substances. This indicates that the peripheral analgesic effects of the studied plant may be caused by the same mechanism of suppression of local peritoneal inflammation and prostaglandin-producing pathways.

The antipyretic effect of the PAAPEE was determined in an animal model. The low-cost and reliable Baker’s yeast-triggered fever model replicates pathogenic fever and can be used to evaluate novel antipyretics. Endogenous pyrogens, including prostaglandins, such as PGE2 and PGI2, IL-1 and IL-6, macrophage protein-1 interferon, and tumor necrosis factor (TNF), are known to cause fever. Brewer’s yeast promotes prostaglandin and TNF synthesis (Stein and Kuchler, 2012). PAAPEE significantly lowered the rectal temperature in rats with yeast-induced pyrexia in a dose-dependent manner when administered orally. After the fourth h of administration, the largest drop was observed with 500 mg/kg of PAAPEE and 20 mg/kg of the traditional medication diclofenac sodium. The effects of PAAPEE antipyretics may be mediated by a reduction in prostaglandin synthesis and the prevention of cytokine release.

Wound-healing is a complicated process that occurs after injury to the skin and soft body tissues. It includes a variety of biochemical reactions aimed at restoring the damaged cell structure to its normal and original form. Typical healing of the wound cascade comprises three overlapping and sequential phases: inflammation, proliferation, and remodeling (Nagar et al., 2016). In the excision wound model done in rats, topical application of the formulated ointments (12.5, 25, and 50% w/w) of PAAPEE topically stimulated wound-healing.

*Panicum antidotale*’s antioxidant capacity was determined utilising a number of *in vitro* antioxidant tests, including DPPH. In nature, antioxidants and free radicals, also known as reactive oxygen species, coexist. A disturbance in this balance may cause damage to cellular components and oxidative stress, which can lead to any disease, including inflammation. Antioxidants prevent cellular damage produced by free radicals and reactive oxygen species by preventing the oxidation of biological components (Mishra et al., 2012; Ullah et al., 2014a). The lower the inhibitory concentration in DPPH assays, the higher the antioxidant activity of the chemical. *Panicum antidotale* contains flavonoids and phenolic chemicals, which function as antioxidants and free radical scavengers and contribute to the plant’s anti-inflammatory properties.

HPLC analysis of PAAPEE revealed its richness in phenolic metabolites and flavonoids. Flavonoids are polyphenolic compounds that exist naturally in the environment and were previously reported to suppress TNF-α, COX, reactive oxygen species, and IL production (Geronikaki and Gavalas, 2006). Flavonoid glycosides suppress the synthesis of IL-1, TNF-α, and NO, resulting in anticancer, and anti-inflammatory effects (Rathee et al., 2009). The antioxidant activity of PAAPEE was assessed using a DPPH free radical scavenging capacity assay. Antioxidants, and free radicals, also known as reactive oxygen species, coexist. Disturbances in this balance may damage cellular components and cause oxidative stress, which can lead to various diseases, including inflammation. Antioxidants shield cells from the harmful effects of reactive oxygen species and free radicals by preventing the oxidation of biological components. The lower the inhibitory concentration in the DPPH assay, the higher the antioxidant activity of the chemical. PAAPEE contains flavonoids and phenolic metabolites, which function as antioxidants and free radical scavengers and contribute to the plant’s anti-inflammatory properties (Ullah et al., 2014a).

Additionally, molecular docking performed on HPLC identified phenolic constituents, and flavonoids in the PAAPEE on 5-LOX, and GSK3*β* that is involved in the wound-healing process, revealed that all identified metabolites exhibited notable activity within the tested enzyme active sites that further ascertained the results of *in vivo* studies.

## 5 Conclusion

The results of the current study demonstrate that PAAPEE possesses wound-healing properties and exhibits anti-inflammatory, analgesic, antipyretic, and antioxidant activities based on various performed *in vivo* studies. These properties are thought to result from numerous phytoconstituents, particularly phenolic components, as revealed by HPLC analysis. Molecular docking studies further supported these findings by identifying the molecular targets and highlighting the activity of the examined metabolites within the active sites of 5-LOX, which is involved in the inflammatory process, and GSK3β, which plays a role in wound-healing process. Meanwhile, molecular docking only gave a preliminary prediction of the probable binding with the receptor that may contribute to activity and this should be followed by *in vitro* examination and preclinical trials. However, it is recommended to standardize the extract, isolate the secondary metabolites and conduct further biological evaluations. Identifying novel and effective wound-healing, anti-inflammatory, antipyretic, and analgesic entities is critical for improving research and application in this field.

## Data Availability

The original contributions presented in the study are included in the article/Supplementary material, further inquiries can be directed to the corresponding authors.
